# Supplementary material for: Machine Learning Accelerates Crystallization for Structure Determination
Source: Angew Chem Int Ed Engl. 2026 May 4;65(25):e1218503. doi: 10.1002/anie.1218503 (PMC13266958; doi:10.1002/anie.1218503)

checkCIF/PLATON report

Structure factors have been supplied for datablock(s) lcz106

THIS REPORT IS FOR GUIDANCE ONLY. IF USED AS PART OF A REVIEW PROCEDURE FOR PUBLICATION, IT SHOULD NOT REPLACE THE EXPERTISE OF AN EXPERIENCED CRYSTALLOGRAPHIC REFEREE.

No syntax errors found. CIF dictionary Interpreting this report

Datablock: lcz106

Bond precision: C-C = 0.0075 A Wavelength=1.54184

Cell:	a=9.9907(1)	b=13.2568(2)	c=18.8672(2)
	alpha=79.971(1)	beta=75.551(1)	gamma=80.568(1)
Temperature:	100 K		

	Calculated	Reported
Volume	2363.77(5)	2363.77(5)
Space group	P -1	P -1
Hall group	-P 1	-P 1
Moiety formula	C38 H41 Ag3 F18 N6 O5	C15 H3 Ag3 F18 N6, C23 H38 O5
Sum formula	C38 H41 Ag3 F18 N6 O5	C38 H41 Ag3 F18 N6 O5
Mr	1327.38	1327.38
Dx,g cm-3	1.865	1.865
Z	2	2
Mu (mm-1)	10.963	10.963
F000	1308.0	1308.0
F000’	1313.79	
h,k,lmax	12,16,23	12,16,23
Nref	10130	9711
Tmin,Tmax	0.174,0.215	0.618,1.000
Tmin’	0.080	

Correction method= # Reported T Limits: Tmin=0.618 Tmax=1.000
AbsCorr = MULTI-SCAN

```
Data completeness= 0.959            Theta(max)= 78.188

                                                      wR2(reflections)=
R(reflections)= 0.0449( 8746)                         0.1253( 9711)

S = 1.056                    Npar= 635
```

The following ALERTS were generated. Each ALERT has the format
test-name_ALERT_alert-type_alert-level.
Click on the hyperlinks for more details of the test.

Alert level C

```
PLAT042_ALERT_1_C Calc. and Reported MoietyFormula Strings  Differ     Please Check
              Calc: C38 H41 Ag3 F18 N6 O5
              Rep.: C15 H3 Ag3 F18 N6, C23 H38 O5
PLAT906_ALERT_3_C Large K Value in the Analysis of Variance ......      3.908 Check
PLAT911_ALERT_3_C Missing FCF Refl Between Thmin & STh/L=    0.600         37 Report
                1  0  0,   0  1  0,   0 -1  1,   1 -1  1,   8  0  1,   0  1  1,
                0  0  2,   1  0  2,  -2 -2  3,   2 -1  3,   2  0  3,   4 15  4,
               -2  8  5,   0  2  6,  -3  1  9,  -6 -2 13,   5 -6 15,  -3 -7 16,
               -2 -7 17,  -3 -6 17,  -2 -6 17,  -3 -5 17,  -2 -5 17,  -1 -6 18,
               -2 -5 18,  -1 -5 18,  -3 -4 18,  -2 -4 18,  -1 -5 19,  -2 -4 19,
                   (    7 More Missing: see the .ckf listing file)
PLAT934_ALERT_3_C Number of (Iobs-Icalc)/Sigma(W) > 10 Outliers ..          1 Check
               -4 -3  5,
PLAT977_ALERT_2_C Check Negative Difference Density on Hc        .      -0.31 eA-3
```

Alert level G

```
PLAT007_ALERT_5_G Number of Unrefined Donor-H Atoms .............          1 Report
              H00D
PLAT083_ALERT_2_G SHELXL Second Parameter in WGHT  Unusually Large       8.34 Why ?
PLAT154_ALERT_1_G The s.u.'s on the Cell Angles are Equal ..(Note)      0.001 Degree
PLAT242_ALERT_2_G Low    'MainMol' Ueq as Compared to Neighbors of       C011 Check
PLAT242_ALERT_2_G Low    'MainMol' Ueq as Compared to Neighbors of       C012 Check
PLAT242_ALERT_2_G Low    'MainMol' Ueq as Compared to Neighbors of       C01A Check
PLAT242_ALERT_2_G Low    'MainMol' Ueq as Compared to Neighbors of       C01D Check
PLAT242_ALERT_2_G Low    'MainMol' Ueq as Compared to Neighbors of       C01I Check
PLAT242_ALERT_2_G Low    'MainMol' Ueq as Compared to Neighbors of       C01S Check
PLAT335_ALERT_2_G Check Large C6 Ring C-C Range C00Z     -C01F           0.15 Ang.
PLAT434_ALERT_2_G Short Inter HL..HL Contact F00P      ..F00V     .      2.80 Ang.
                                                1-x,2-y,1-z  =      2_676 Check
PLAT720_ALERT_4_G Number of Unusual/Non-Standard Labels ..........        105 Note
              Ag01    Ag02    Ag03    F004    F005    F006    F007    N008
              F009    F00A    F00B    O00C    O00D    H00D    O00E    O00F
              F00G    O00H    N00I    F00J    F00K    N00L    N00M    N00N
              F00O    F00P    F00Q    F00R    N00S    F00T    F00U    F00V
              F00W    C00X    C00Y    H00A    H00B    C00Z    C010    C011
              C012    C013    C014    C015    H015    C016    C017    C018
              C019    C01A    C01B    H01A    H01B    C01C    C01D    C01E
              H01C    H01D    H01E    C01F    C01G    C01H    C01I    C01J
              H01F    H01G    C01K    H01H    H01I    C01L    H01J    H01K
              C01M    C01N    H01L    H01M    C01O    H01N    H01O    C01P
              H01P    H01Q    H01R    C01Q    H01S    H01T    C01R    H01U
```

```
              H01V    H01W    C01S    C01T    H01X    H01Y    C01U    H01Z
              C01V    H01     Ha      C01W    Hb      C01X    Hc      C01Y
              Hd
PLAT912_ALERT_4_G Missing # of FCF Reflections Above STh/L=  0.600        382 Note
PLAT913_ALERT_3_G Missing # of Very Strong Reflections in FCF ....          3 Note
               -2 -2  3,   2 -1  3,   0  2  6,
PLAT941_ALERT_3_G Average HKL Measurement Multiplicity ..........         2.6 Low
PLAT969_ALERT_5_G The 'Henn et al.' R-Factor-gap value ..........       3.995 Note
              Predicted wR2: Based on SigI**2  3.14 or SHELX Weight 11.87
PLAT978_ALERT_2_G Number C-C Bonds with Positive Residual Density.          0 Info
```

```
   0 ALERT level A = Most likely a serious problem - resolve or explain
   0 ALERT level B = A potentially serious problem, consider carefully
   5 ALERT level C = Check. Ensure it is not caused by an omission or oversight
  17 ALERT level G = General information/check it is not something unexpected

   2 ALERT type 1 CIF construction/syntax error, inconsistent or missing data
  11 ALERT type 2 Indicator that the structure model may be wrong or deficient
   5 ALERT type 3 Indicator that the structure quality may be low
   2 ALERT type 4 Improvement, methodology, query or suggestion
   2 ALERT type 5 Informative message, check
```

Validation response form

Please find below a validation response form (VRF) that can be filled in and pasted into your CIF.

```
# start Validation Reply Form
_vrf_PLAT042_lcz106
;
PROBLEM: Calc. and Reported MoietyFormula Strings  Differ     Please Check
RESPONSE: ...
;
_vrf_PLAT906_lcz106
;
PROBLEM: Large K Value in the Analysis of Variance ......      3.908 Check
RESPONSE: ...
;
_vrf_PLAT911_lcz106
;
PROBLEM: Missing FCF Refl Between Thmin & STh/L=    0.600         37 Report
RESPONSE: ...
;
_vrf_PLAT934_lcz106
;
PROBLEM: Number of (Iobs-Icalc)/Sigma(W) > 10 Outliers ..          1 Check
RESPONSE: ...
;
_vrf_PLAT977_lcz106
;
PROBLEM: Check Negative Difference Density on Hc        .      -0.31 eA-3
RESPONSE: ...
;
# end Validation Reply Form
```

It is advisable to attempt to resolve as many as possible of the alerts in all categories. Often the minor alerts point to easily fixed oversights, errors and omissions in your CIF or refinement strategy, so attention to these fine details can be worthwhile. It is up to the individual to critically assess their own results and, if necessary, seek expert advice.

PLATON version of 17/09/2025; check.def file version of 17/09/2025

---

duplicate check

No duplication found

Datablock lcz106 - ellipsoid plot

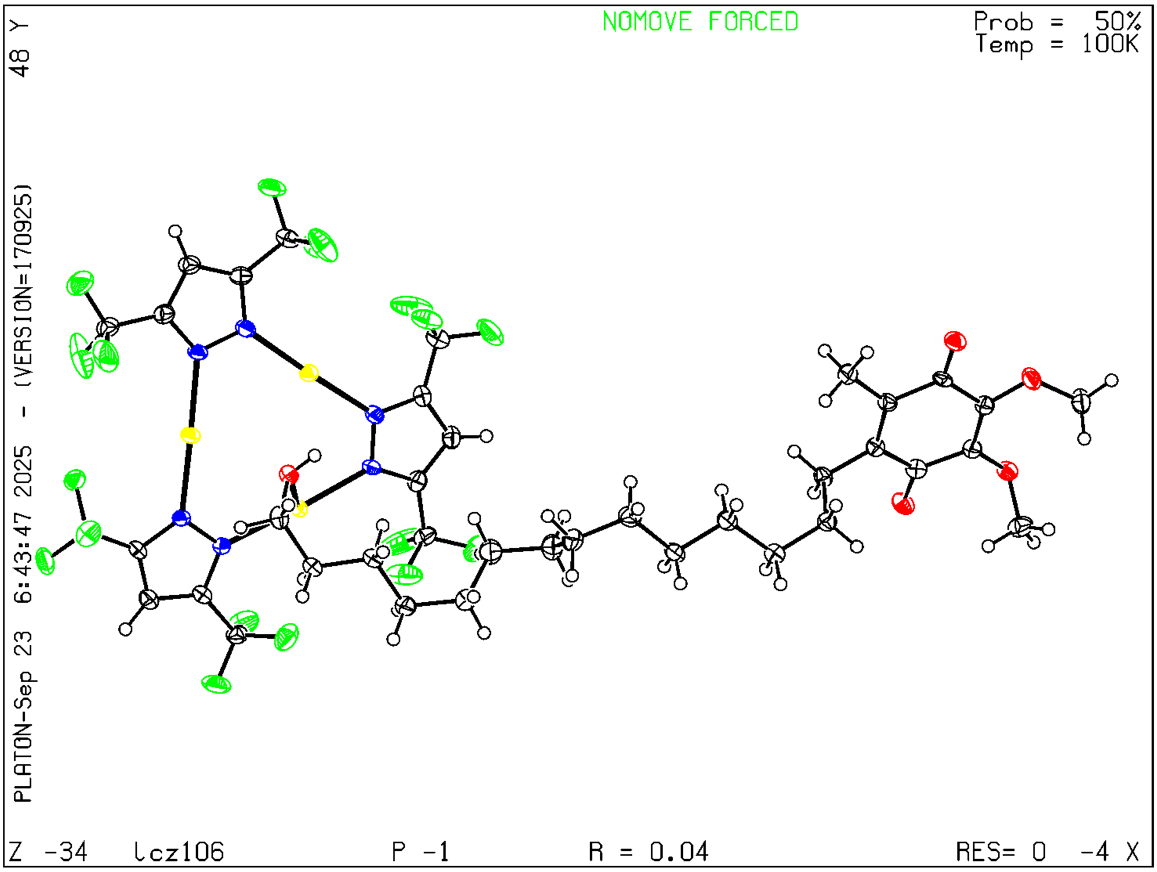

Supplement: Supplementary file 2 — Supporting File 2: anie72462‐sup‐0002‐DataFile.zip. [file ANIE-65-e1218503-s002.zip › supplementary_materials_crystallographic_data submit.zip!/cif_and_checkcif(1-114)/Ag3Pz3_106/Ag3Pz3_106_checkcif.pdf]
